# The Effectiveness of Digestate Use for Fertilization in an Agricultural Cropping System

**DOI:** 10.3390/plants10081734

**Published:** 2021-08-22

**Authors:** Modupe Olufemi Doyeni, Urte Stulpinaite, Ausra Baksinskaite, Skaidre Suproniene, Vita Tilvikiene

**Affiliations:** Lithuanian Research Centre for Agriculture and Forestry, LT-58344 Kedainiai, Lithuania; urte.stulpinaite@lammc.lt (U.S.); ausra.baksinskaite@lammc.lt (A.B.); skaidre.suproniene@lammc.lt (S.S.); vita.tilvikiene@lammc.lt (V.T.)

**Keywords:** nitrogen use efficiency, grain yield, grain quality, soil chemical quality, digestates

## Abstract

The need to find and maximize the use of alternative sources of nutrients for plants and soil environment have been on the forefront of research in sustainable agriculture. These alternatives have to be affordable, accessible, reproduceable, and efficient to compete with established inorganic fertilizers while at the same time reduce any potential negative impacts on the environment. We aimed to evaluate the effectiveness of digestate fertilization in an agricultural system over a period of three years. The digestate utilized in the study consisted of animal waste-based digestates, namely pig manure digestate, chicken manure digestate, and cow manure digestate, and were compared with synthetic nitrogen fertilizer. Every year, the digestate and the synthetic nitrogen fertilizer were split applied at the rate of 90 and 80 kg N ha^−1^. The soil chemical composition after three years of fertilization showed a slight decrease, significantly different nitrogen and carbon changes while phosphorus and potassium were significantly higher in the digestate treatments. The third year of digestate application showed higher grain yield than previous years and the yield from the digestate treatments were significantly different from the synthetic nitrogen fertilizer. The nitrogen use efficiency for the three years was in the range of 20–25 percent in the digestate treatments, with a strong correlation between the nitrogen use efficiency and the grain yield. There were varied results in the grain quality and straw quality in the digestate and synthetic nitrogen fertilizer with no clear trend observed. Our results showed a relatively high potential of animal waste digestates over the short to mid-term use with a positive result obtained in comparison to synthetic nitrogen fertilizer under favorable climatic conditions.

## 1. Introduction

The demand on the agricultural systems has increased tremendously in recent times occasioned by the demand from a consistently growing human population amidst limited land resource. These demands are premised but not limited to these three challenges—food security, income for farmers, and providing a safer environment. To meet the demands enumerated, agricultural management practices such as organic farming, agroecological methods, and environmentally friendly methods are being employed to meet and surpass these targets in a sustainable way from short to long term. One of the researched and encouraged methods of agricultural management is the application of digestates to agricultural soils [1,2,3,4]. In the EU, an estimated 180 million tons of anaerobic digestate are produced per year, most of which is used as organic fertilizer [5]. The treated anaerobic wastes are products from different organic feedstocks sources, which include wastewater treatment, plant sludge (primary and secondary sludge), agri-food industry waste (part of municipal solid waste including fruit and vegetable by-products, canteen waste, kitchen waste), green waste (waste from shearing of grass, sheets), animal waste (swine, dairy manures), and food-waste (animal fats, used cooking oils, restaurant vats for degreasing [6].

Digestate is one of the resulting products generated from biogas systems and are rich in nutrients. Digestate has the capacity to compete favorably with inorganic fertilizers for better crop productivity, yield, and enhancement of soil health [1,7]. One of the benefits of utilizing digestate is the higher nutrient content than their respective feedstock. Although, from the resulting digestate, a considerable amount of nitrogen (N) in the ammonium form is emitted during the anaerobic digestion process, while carbon (C) is also removed as methane and carbon dioxide. However, a good proportion of nutrients such as N, phosphorous (P), and potassium (K) are retained [8]. The mineral contents and characteristics of digestate depend, most of the time, on the characteristics of the substrate and the modality of digestion [9]. These defined attributes, such as organic matter content, NH_4_, the C/N ratio, and N content present in the different substrates or feedstocks that form the digestates, will show differences in efficiencies and productivity in plants and soils, therefore making it the main source of this research.

Digestate hold multiple functions in their beneficial roles to both the soil and the plants/crops. In the first instance, digestate is known to have fertilizing attributes that help in the productivity of the plants due to the availability of important nutrients necessary for plant growth. Secondly, their influence on soil health cannot be overemphasized as they play huge roles that promote soil efficiency through nutrient cycling in the soil, carbon transformation, and soil structure maintenance [10]. The application of digestate in the field could have fewer shorter-term results due to the slow mineralization rate or action from microbes [3]. Nitrogen is an essential element for plant growth and soil microbial activity and is the nutrient taken up in the largest amount by plants and the most common limiting factor for plant growth [11,12].

The contribution of digestate to the N availability in the soil presents an important argument for their application. Digestate is particularly rich in ammonium nitrogen (NH_4_-N), a form of N that is readily available for uptake by plants [13]. The ratio of the mineral N fraction to the total N content is an important indicator of its impact on the N cycle and transformation in soil during plant growth. However, while it has become necessary to improve crop productivity through the use of N based fertilizer, the need to increase nitrogen use efficiency (NUE) in the quest for better agronomic and environmental result must be taken into consideration.

Good productivity and yield of crops can be achieved if the right amount of N inputs are introduced into the soil system. Excess N from over-application in the soil environment can subsequently lead to toxicity, leaching, ammonia volatilization, and environmentally harmful N_2_O emissions. Hence, the need for N management is required to manage and maximize the efficiency of plants to use the applied N. Improvements in NUE are aimed at decreasing N loss through a decrease in the usage of N fertilizers (digestate inclusive) encouraged by plant uptake. Thus, there is a need to identify strategies and agricultural practices to increase NUE. Nitrogen use efficiency for cereal production can be defined from the perspective of three different approaches: Agronomic efficiency, environmental efficiency, and the economic efficiency [14]. However, from this study, consideration was taken from the agronomy and the environment standpoint where NUE was determined as the ratio between the amount of N utilized by the crop and the amount of N fertilizer applied. This correlated with the way required information on NUE are sourced and used in relation to the utilization of additional N applied to an agricultural production system in a country or region [14,15]. The continued use of digestate in agricultural systems has shown both positive and negative environmental attributes as reviewed [2]. However, their roles in improving crop production and crop yield tend to show variation depending on different factors such as climatic conditions, soil properties, composition of digestate, crop species, and time dependent. In essence, while their influence has been established, others have reported that their application has resulted in lower yields when compared with inorganic fertilizers [16]. The observed variability of the wheat response to fertilization using digestate is due to the interaction of numerous factors, including dose and type of substrate [17]. Nonetheless, our knowledge about the effects of digestate on the yield and quality of varieties of cereals are still limited. In particular, regarding plant responses to fertilization using digestate, there are no recognized relationships between grain and nutrient content. Understanding this relationship is important since cereals are a main source of protein and mineral nutrients in the human diet. Their supporting role in food production as sources of organic material for soil health and carbon sequestration cannot be waived in the quest for food security. Cereal production has continued to receive global attention as FAO had forecasted 2817 million tons of cereal production for 2021 [18]. To achieve this milestone, their yield and nutritional contents are necessary to be maintained and improved upon with the utilization of different agricultural organic amendments (digestate) to supplement the use of inorganic fertilizers.

The aim of this study was to evaluate the effectiveness of the application of different animal waste-based digestate on the cultivation of different cereals with reference to synthetic nitrogen fertilizer. It was hypothesized that (i) the soil chemical composition in terms of C and N would not be negatively impacted, (ii) the digestate application to soil would improve crop yield and productivity, and (iii) the NUE would increase with the years of digestate application.

## 2. Results and Discussion

### 2.1. The Changes in Soil Quality after Digestate Treatment

The research was aimed to analyze the changes in soil quality after three years of digestate application in traditional cropping system. It was found that N content in the soil changed minimally with a slight decrease in the control (not fertilized) and the fertilized treatment of pig manure digestate in comparison to the increase observed in treatments, fertilized with synthetic mineral nitrogen fertilizers, chicken, and cow manure digestates (Table 1). There was no significant difference between treatments that presented a N increase while pig manure digestate presented a decrease in the N content. The three different digestates showed slight but different changes after three years of digestate application. The differing N changes can be explained by the different qualities of the used feedstock, therefore presenting a strong determinant in the chemical properties and actions of the digestate [19,20]. The result also supported the general view that the digestate application does not cause any significant changes in the total N [11,21,22] despite three-year application. This was very positive for the digestate application to soil for such a period of time. Thus, their application under the right dose and management system including that of synthetic mineral nitrogen supported the reported studies [20,23], where the supply of N consistently met the demand placed on the plants and the soil and further reduced nitrate leaching potential. Furthermore, the high proportion of NH_4_-N present in the digestate has the potential to increase NH_4_-N content of the amended soil, rapidly nitrified [23] and with a higher ability of N loss arising from volatilization especially from pig manure digestate, which has a lower organic matter content and C:N ratio.

Three-year digestate application did not increase carbon content (Table 1) in the soil. As it was expected, the treatments fertilized with pig and chicken manure digestate presented even higher carbon decrease in the soil. Barłóg et al. [24] and Möller et al. [25] had reported earlier that digestate treatment did not significantly influence soil organic carbon with no substantial increase. In contrast, our study indicated a slight decrease in soil carbon after three years of application. In addition, the result observed in our study presented a considerably lower carbon content decrease in synthetic nitrogen fertilizer compared with the digestate treatments. This trend was reported earlier [1], although the digestate were applied for a longer period of seven years. Furthermore, investigation of the changes in carbon content after three years showed significant differences in the three digestate treatments, explained by the distinct characteristics of their primary feedstocks and the assumption of the influence of the lower dry matter content in digestate. Another expected reason could be the increased soil microbial activity, which was assumed to lead to more intensive mineralization of organic matter. The carbon served as energy sources for soil microorganisms to flourish and their availability must have resulted in CO_2_ emissions in support of an earlier study [4]. Digestate derived from animal waste seem to accumulate less carbon without recourse to the number of years of fertilization.

The highest changes were obtained in potassium content in the soil. The potassium concentration after the three years experiment decreased in control plots as well as in those fertilized with synthetic mineral fertilizer while the significant increase was obtained in plots, fertilized with all types of digestate. Fertilization with pig, chicken, and cow manure digestate increased potassium content by 22.33, 65.67, and 67.00 mg kg^−1^, respectively (Table 1). The increase observed in our study lays credence to previous studies of higher phosphorous concentration after digestate application [8,26]. The availability of phosphorus in readily available forms and the strong potential to increase their concentration makes digestate an important option for consideration when there is a need to supplement soils with phosphorus as missing macronutrients. Phosphorus content increased in all treatments except for the one fertilized with chicken manure digestate (Table 1). Despite the fact that chicken manure digestate is rich in phosphorus, the amount of applied digestate was relatively low, because the rate was calculated according to the total N content. That could be one of the main factors as to why phosphorus content decreased in this treatment. In summary, the use of digestate fertilization increased soil quality by increasing potassium and phosphorus (except chicken manure digestate) content in the soil.

There were no significant differences (*p* < 0.05) in the pH of the digestate and the synthetic mineral nitrogen, although there was a decrease in pH over the course of the three-year digestate application. The pH of the amended soil was lower than the unamended treatment. The pH of the soil after three years of application resulted in lower pH on the basis of the use of N sources contained in the fertilizers containing ammonium-N. As the NH_4_-N in fertilizers undergoes nitrification (conversion of ammonium to nitrate in soils by bacteria), hydrogen (H^+^) is released, which can increase acidity. As the percentage of ammonium increases in a given fertilizer, the acidifying potential will also be increased, thus reducing the pH. However, the decrease in pH was small and not of significant impact to the growth and productivity of plant and soil health going by the management and monitoring of the N content in the digestate. It is, however, debatable whether the application of digestate could influence the soil pH significantly in the long term, since the applicable amounts are usually restricted by nitrogen content.

### 2.2. Grain Productivity

**Figure 1:**
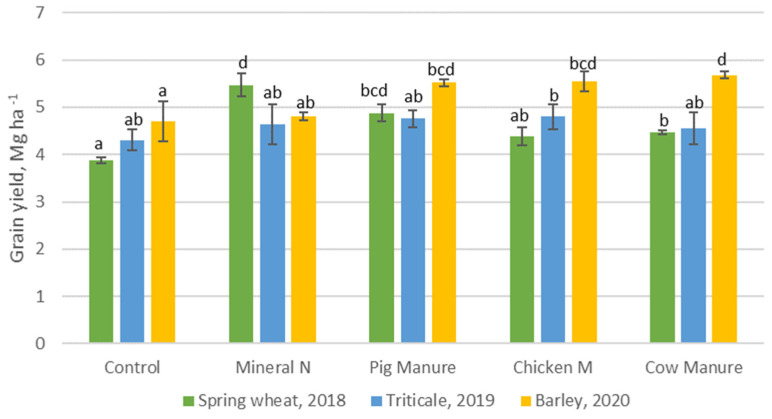
Grain yield of spring wheat (2018), triticale (2019), and barley (2020), fertilized with synthetic mineral nitrogen fertilizer and different types of digestate. The columns with different letters show the significant differences between treatment for each crop at *p* < 0.05.

The grain productivity was analyzed in all three years of the experiment. In the first year (2018) of the experiment, the highest grain yield was obtained in the treatment fertilized with synthetic mineral nitrogen fertilizer and the treatment fertilized with pig manure digestate (Figure 1) while the other treatments did not present higher grain productivity, compared to the control plots. In 2019 (the second year of the experiment), the extremely dry weather conditions resulted in lower availability of fertilizers for plants and there were no significant differences (*p* < 0.05) obtained between the different treatments. In the third year of the experiments (2020), the benefit of digestate fertilizers was obtained. In this year, all crops fertilized with the different types of digestate produced significantly higher grain productivity, compared to the non-fertilized ones and the synthetic mineral nitrogen fertilizers. This may conclude that three years of consistent and proper management of digestate application was favorable to increase soil fertility, which influences effective crop production. The effective crop production can be noted by results of equal or better yields than synthetic nitrogen fertilizer with a longer period of digestate application. This aligns with studies from [2,22,26]. Chantigny et al. [27] also reported that digestate application produced higher yields as the necessary nutrients needed for growth and yield were provided for plant uptake and soil fertility. Longer-term digestate application ranging from three to six years also resulted into higher yields in other studies [1,13,27,28,29,30].

### 2.3. Grain Nutritional Quality

The effect of fertilization on the selected grain quality depended on the tested crop and the fertilizer treatments (Table 2). The protein content is very important and helps to determine the effectiveness of the digestate as N source for the quality of grain. Our study showed that all the protein content in the cereals observed in the digestate and synthetic nitrogen treatments for the three years exceeded 11.5%. This range particularly signifies high-value, protein-rich content important for food production while grain below 11.5% generally finds its way into the stock-feed market [29]. Furthermore, the protein contents in all the treatments were significantly higher (*p* < 0.05) than the unamended treatment (control) for the three years of different crop cultivation. However, between the amended plots, synthetic N had a protein content that was significantly different from the three digestate treatments in all the crops and higher in spring wheat and triticale. The grain protein content helped to show the plant nitrogen intake, and the result of our study indicated the differing N intake between the digestate treatment and the synthetic mineral nitrogen. Nitrogen uptake increases with the application of fertilizer N due to increases in crop yield, and to a lesser extent, increases in grain protein [30]. Hence, with the same application rate of N (170 kg N), this resulted in increased grain protein content, evidencing an optimization of NUE and an assumption of a moderate loss of N through N_2_O emission.

The grain density in the digestate and synthetic N treatments were higher in the three cereals compared to the control, which was an indication of the N sources added. This aligns with previous findings where the nitrogen content is consistently positively correlated with grain density [31].

### 2.4. Chemical Content in Straw for Straw Quality

**Figure 2:**
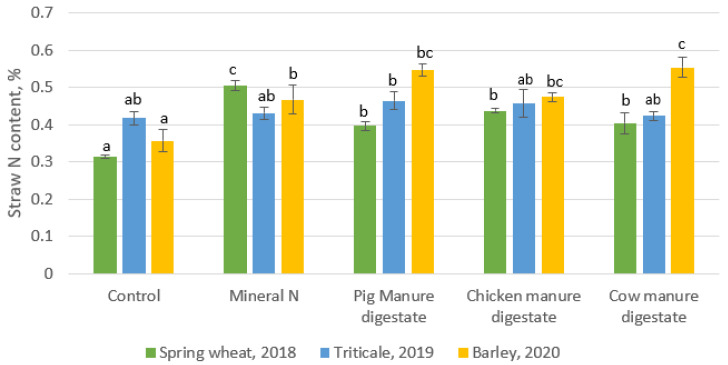
Straw Nitrogen content for 3 years. The columns with different letters show the significant differences between treatment for each crop at *p* < 0.05.

**Figure 3:**
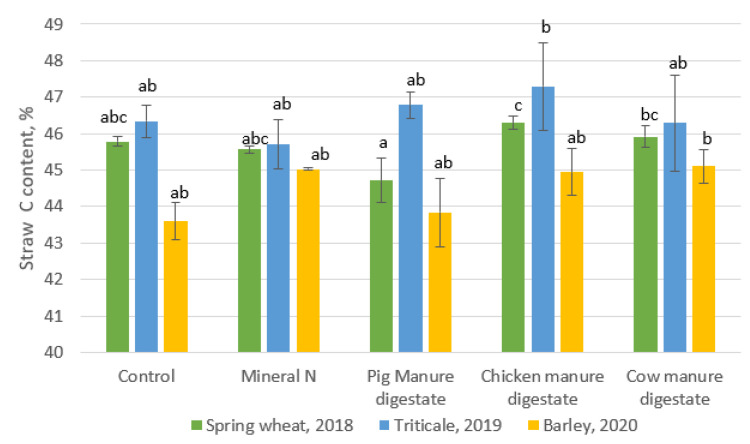
Straw Carbon content for 3 years. The columns with different letters show the significant differences between treatment for each crop at *p* < 0.05.

The straw composition is a major consideration in the determination of the influence of amendment on plant productivity. In determining the straw quality in terms of the N and C content in the dried straw, the treatments had different varying effects on the straw selected content of the three crop straws. The result showed that nitrogen content in straw (Figure 2) obtained from the synthetic nitrogen fertilizer was highest in spring wheat cultivated in the year 2018. The treatment with synthetic nitrogen was significantly different from other treatments in 2019 (straw of triticale). In addition, for the year 2020 (barley), pig manure digestate and cow manure digestate had a higher N content in straw in comparison to the other treatments. For the C content in the straw (Figure 3), in the year 2018, there was no significant differences in all the treatments while in the year 2019, the C content in the straw treated with chicken manure digestate was significantly different from other treatments. For the year 2020, cow-manure-treated straw was significantly different with a higher C content than the other treatments.

### 2.5. Nitrogen Use Efficiency

**Figure 4:**
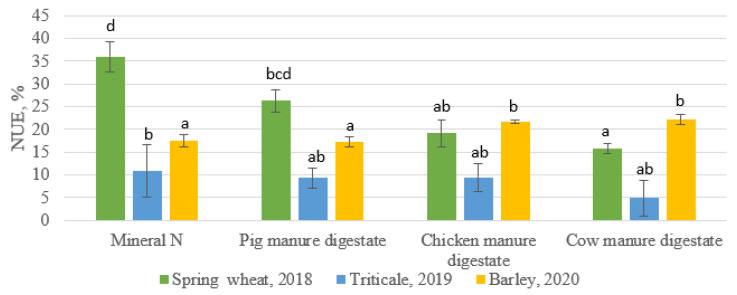
Nitrogen Use Efficiency of organic ammendments. The columns with different letters show the significant differences between treatment for each crop at *p* < 0.05.

The Nitrogen use efficiency (NUE) for the four treatments were calculated for the three years to estimate whether such application led to nitrogen efficiency or not over long-term use and an indication to improve their implication. Generally, an estimated 33% NUE was envisaged by different studies [32,33,34]; however, efforts are being put in place through different agricultural practices to improve the NUE ratio. In this study, an approximate 35% NUE was observed in synthetic nitrogen in the first year compared to the lowest NUE of 16% observed in cow manure digestate (Figure 4). There was a significant drop in NUE efficiency in synthetic nitrogen fertilizer from 2018 when compared to 2019. The NUE in synthetic nitrogen fertilizer was significantly different from the NUE in the digestate treatments in the second year of amendment. A lower NUE was observed in the year 2019 across all the treatments, with the assumption that there must have been N loss through nitrate leaching or unfavorable climatic factors in consonance with a previous study [35]. The short-term benefit of the low NUE will result in more organic N being stored in the soil and available for plant uptake in the subsequent year. For the year 2020, there was a slight increase in NUE in all treatments, although NUE rates were lower in synthetic nitrogen fertilizer and pig manure digestate when compared to the year 2018—the first year of application. The NUE increased in chicken manure digestate and cow manure digestate in comparison to the first year of application, although it should be noted that the NUE rates under this treatment including pig manure digestate fall below 25% NUE but were well above the NUE rate in the second year of fertilization. This showed an overall effective utilization of the available N nutrient ratio as the period of fertilization progressed. It is noteworthy that the yearly use of N fertilizers coupled with the split application of fertilizers ensured an improvement of NUE for the cereal growth in the subsequent two years, in agreement with previous results [35,36]. There could have been other sources of losses such as N_2_O emissions probably due to the open nature of an agricultural system [4,37]. We also opined that volatilization of N would be low and might not have contributed to the lower NUE in all years as the mode of application of the digestates was by direct injection [15,38].

### 2.6. Relationship between Nitrogen Use Efficiency and Crop Yield

**Figure 5:**
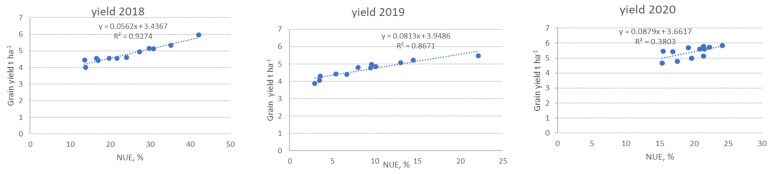
Relationship between grain yield and NUE for the years 2018, 2019, and 2021.

In a bid to estimate how the NUEs are related to crop yields for the three years under study, the relationship between the two factors were determined under the N application rate of 170 kg N ha^−1^ (Figure 5). In the years 2020 and 2019, NUE had a very strong correlation of R = 0.93 and R = 0.90, respectively, while for the year 2018, the relationship was R = 0.63. Most organic compounds like amino acids, proteins, nucleic acids, and compounds of secondary plant metabolism have N as their important make-up [15,39], making it a pathway for crop productivity and yield. The efficiency of N uptake and use relative to the production of grain requires that the processes associated with absorption, translocation, assimilation, and redistribution of N operate effectively [34,40]. The addition of N into the soil caused the retention of N in the upper soil profile, which invariably increased the NUE arising from greater N uptake and resulting in less or decreased N in soil after harvest. Hence, the uptake of N in higher amounts by plants and its transfer to grain is crucial for increasing yields as we progress in the year with subsequent amendment.

## 3. Materials and Methods

### 3.1. Experimental Site

The experimental study was carried out in the fields of the Lithuania Research Centre for Agriculture and Forestry (55°40′ N, 23°87′ E) for 3 years (2018 to 2020 growing seasons). The soil of the experimental fields was Endocalcari-Epihypogleyic Cambisol, and the soil chemical composition taken at a depth of 0–20 cm from the start of the experiment (year 2018) is shown in Table 3. The experiment was set up at the start with the Spring wheat (*Triticum aestivum*) cultivar “*Collada*” (Einbeck, Germany), in the second year with Spring triticale, a hybrid between the wheat and rye cultivar “*Milkaro*” (Koscian, Poland); and in the third year, the spring barley (*Hordeum vulgare L*.) cultivar ‘*Ema DS*’ (Akademija, Lithuania). The sowing rate was 270 kg ha^−1^ (spring wheat), 250 kg ha^−1^ (spring triticale), and 220 kg ha^−1^ (spring barley). Seeds were sown on 19 April 2018, 16 April 2019, and 16 April 2020, respectively. The cultivation period was from April to September for the three (3) years.

### 3.2. Experimental Design

The field experiment was established in a complete randomized design with 5 treatments in three replicates each tested for three years. Each treatment plot was 30 m^2^ (3 m × 10 m). The experimental treatments were as follows: Unfertilized (control), fertilized with the synthetic nitrogen fertilizer, pig manure digestate, chicken manure digestate, and cow manure digestate. The 170 kg of N ha^−1^ presented in liquid form of the digestates were split fertilized at an application rate of 90 and 80 kg N ha^−1^. For each digestate application, the rate of digestate was calculated according to its content of total nitrogen. For chemical analysis, soil samples were collected before the first-year cultivation and after the third-year harvest (after 29 months) from the start of the experiment. The determination of soil acidity (pH) was made in a 1:5 (vol^−1^) soil suspension in the 1M KCl solution [40]. Soil mobile potassium (K_2_O), mobile phosphorus (P_2_O_5_), mobile calcium (Ca), and mobile magnesium (Mg) were determined using an ammonium lactate–acetic acid extraction, as described by Egner, Riehm, and Domingo [40]. The Kjeldahl method was used to determine the total nitrogen (Ntot) in spring wheat, spring triticale, and barley grain and straw. The grain density and protein were determined using the InfratecTM 1241 device to examine the grain quality indicators. Large samples were screened with an Infratec instrument for testing impurities and separated very fine impurities so that the grains in question do not exceed 1% garbage impurities. All chemical analyses were performed in triplicate (*n* = 3). Crop yield was determined by weighing. Spring wheat, spring triticale, and barley grain and straw from each experimental plot were collected and weighed.

### 3.3. Determination of Digestate Chemical Composition

The digestates were obtained from agricultural biogas plants at three locations in Lithuania with the treatments used serving as their primary feedstocks. The digestates were spread on the soil surface without injection on the treatment plots. The chemical analysis of the digestates used in the field experiments for each year was carried out. A flame photometer (FP) (Sherwood, Cambridge, UK) was used to determine mobile potassium K; a UV-VIS Spectrophotometer (Shimadzu, Duisburg, Germany) for mobile phosphorus P; total nitrogen (Ntot) was determined using the Kjeldahl nitrogen distiller method, while Electrical conductivity was determined using a thermo Orion stara2150 star A215Benchtop conductivity meter. For each digestate application, the rate of digestate was calculated according to its content of total nitrogen. The digestate parameters are shown in Table 4.

### 3.4. Weather Conditions

**Figure 6:**
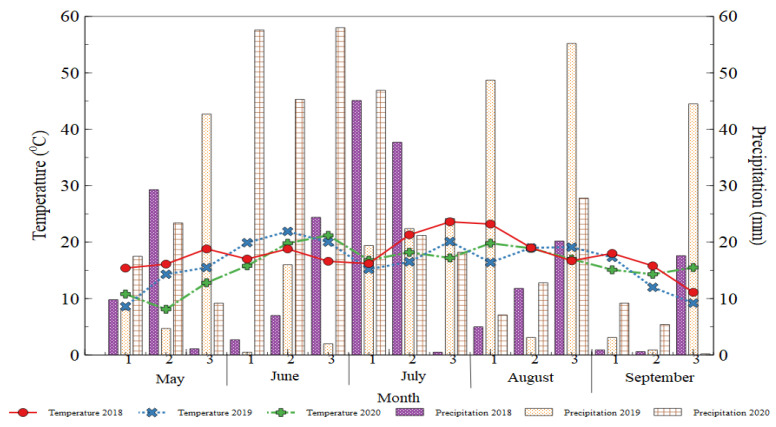
Meteorological conditions for the growing seasons of 2018, 2019, and 2020. 1, 2, 3—represents the average temperature and precipitation taken in 10 days.

Meteorological conditions showing the average air temperatures during the growing season (May–August) of 2018, 2019, and 2020 were 17.48 °C, 16.3 °C, and 16.1 °C, while the long-term average was 15.5 °C. The total precipitation recorded in 2020 was higher and reached 337.4 mm compared to the first year of the experiment (213.7 mm) in May–September. On the contrary, the year 2019 witnessed dry spells with average temperatures of 25.5 and 25.8 °C, respectively (Figure 6). There were dry conditions in the year 2019 compared to the other years. (Lithuanian Hydrometeorological Service- Dotnuva data under the Ministry of Environment data, http://www.meteo.lt/, accessed on 12 July 2021).

### 3.5. Calculation of Nitrogen-Use Efficiency (NUE)

Nitrogen use efficiency (NUE) is the fraction of applied nitrogen that is absorbed and utilized by the plant. In a bid to find a balance between the N inputs and outputs and prevent a negative influence in the application of N-derived fertilizer, the NUE was determined for the significance of their mid- to long-term fertilizer use. Nitrogen use efficiency (NUE) has been defined in several different ways in the literature and the methods to calculate it differ significantly [14,15,39]. The NUE is commonly measured by establishing treatments with and without applying N while taking into consideration the N content in the treatments applied. Therefore, in this study, NUE was calculated by using an output/input ratio according to the methodology described below [15,39].

Nitrogen Use Efficiency (NUE) = (NF) − (NC)/R

where:

NF = total crop N uptake (cereal) from fertilized plots.

NC = total crop N uptake (cereal) from unfertilized plots.

R = rate of fertilizer N applied.

NUE = Nitrogen Use Efficiency.

### 3.6. Statistical and Numerical Analyses

The observed data were statistically processed using SAS 9.4 software. The Duncan Multiple range was applied to determine significant differences between means at an alpha level of 0.05 to determine the influence of the treatments.

## 4. Conclusions

The proper management and use of digestate in agricultural system hold huge potential when its individual feedstock is properly digested in the biogas plants and applied efficiently to agricultural fields in terms of mode of application, split fertilization, and time of application. Based on our research, the properly applied digestate had minimal influence with no negative effects on soil chemical properties after three years of application. The consistent use of digestate as a source of N in soil continued to meet the demand of plants and other critical soil factors when other noticeable means of N loss are minimized, as no significant loss of N was observed over the three years of application. The crop productivity in terms of grain yield and grain quality from the three types of digestate applied showed similarities in output, with some levels of better efficiency when compared with synthetic N fertilizer. The nitrogen use efficiency in the digestate showed an effective utilization of available N as the period of application progressed, with chicken manure digestate and cow manure digestate showing higher NUE when compared to the synthetic nitrogen fertilizer. Digestate as a source of N, when consistently added in a sustainable way, resulted in an increased NUE and a better yield going by their strong interaction displayed at the last two years of digestate application. The mid- to long-term fertilization of digestate in agricultural soil is most beneficial to crop productivity as the quality, quantity (yield), and environment are not in any way compromised.

## Figures and Tables

**Table 1:** The changes of soil chemical composition after three years of digestate application.

Treatment	N, %	C, %	K_2_O (mg/kg)	P_2_O_5_ (mg/kg)	pH
Control	−0.010 c	−0.09 ab	−5.17 c	26.00 a	−0.59 a
Synthetic nitrogen fertilizer	0.004 ab	−0.06 a	−4.17 c	13.67 a	−0.46 a
Pig manure digestate	−0.001 bc	−0.15 b	22.33 b	13.33 a	−0.48 a
Chicken manure digestate	0.013 a	−0.25 c	65.67 a	−9.33 b	−0.49 a
Cow manure digestate	0.0003 abc	−0.04 a	67.00 a	22.33 a	−0.44 a

The columns with different letters show the significant differences between treatment for each crop at *p* < 0.05.

**Table 2:** Grain quality of spring wheat (2018), triticale (2019), and barley (2020), fertilized with synthetic mineral nitrogen fertilizers and different types of digestate.

Treatment	Protein (%)			Density (g dm^−3^)		
	Spring wheat	Triticale	Barley	Spring wheat	Triticale	Barley
Control	10.6 a	11.73 a	10.43 a	81.03 a	62.36 abc	61.4 a
Synthetic nitrogen fertilizer	14.5 d	13.33 d	12.4 ab	82.46 c	63.13 abc	63.03 bcd
Pig manure digestate	14.13 bcd	12.7 b	12.16 bcd	81.9 6bc	63.76 bc	63.11 bcd
Chicken manure digestate	14 ab	12.6 b	13.03 cd	81.9 bc	63.86 c	61.87 ab
Cow manure digestate	12.93 ab	12.23 c	12.76 d	81.93 bc	63.83 bc	63.77 d

The columns with different letters show the significant differences between treatment for each crop at *p* < 0.05.

**Table 3:** Selected physico-chemical properties of the soil (0–20 cm layer).

	pH	P_2_O_5_ (mg/kg)	K_2_O (mg/kg)	Organic C (%)	Ca (mg/kg)	Mg (mg/kg)	N (%)
Soil chemical composition	7.03	134.00	142.67	1.30	4139.33	947.33	0.14
Standard deviation	0.15	6.56	15.04	0.15	955.53	258.66	0.02

**Table 4:** Digestate physico-chemical parameters.

Indicator	Pig Manure Digestate			Chicken Manure Digestate			Cow Manure Digestate		
	2018	2019	2020	2018	2019	2020	2018	2019	2020
pH	8.20	9.00	8.6	7.60	9.80	8.80	8.10	8.30	8.40
Organic matter (%)	1.59	3.42	3.20	3.65	3.13	3.27	1.14	5.04	4.2
Total Nitrogen (%)	0.16	0.51	0.33	0.62	0.51	0.59	0.26	0.34	0.32
P_2_O_5_ (%)	0.15	0.12	0.13	0.21	0.15	0.18	0.14	0.11	0.16
K_2_O (%)	0.13	0.58	0.38	0.24	0.23	0.23	0.13	0.33	0.21
Electrical Conductivity (mS m^−1^)	396	454	402	370	428	410	346	287	312

P_2_O_5_, phosphorus pentoxide; K_2_O, potassium oxide.

## Data Availability

Not Applicable.
